# Communicating Air Quality Index Information: Effects of Different Styles on Individuals’ Risk Perception and Precaution Intention

**DOI:** 10.3390/ijerph181910542

**Published:** 2021-10-08

**Authors:** Yuheng Wu, Lin Zhang, Jilong Wang, Yi Mou

**Affiliations:** School of Media & Communication, Shanghai Jiao Tong University, Shanghai 200240, China; wuyuheng@sjtu.edu.cn (Y.W.); zhanglinnews@sjtu.edu.cn (L.Z.); jlwang@sjtu.edu.cn (J.W.)

**Keywords:** air quality index, smog, valence, third-person effect, risk perception, precaution intention

## Abstract

Air Quality Index (AQI) is information about atmospheric pollutants, which is essential for governments to inform the public about the current air quality and potential health risks. By analyzing the AQIs from 11 countries (regions), we discovered considerable variations in the design of AQI information, which may open up room for unintended interpretation from the public. Therefore, as an attempt to address the inefficiency of some common styles of AQI information in promoting the public’s precaution against bad air and better design such information, an online experiment with a 2 (descriptor: neutral vs. negatively valenced) × 2 (target groups in AQI warning messages: vague vs. specific) factorial design was conducted to test the effects of such information on individuals’ risk perception and precaution intention. The results indicated that AQI information with a neutral descriptor was associated with lower self-risk perception and precaution intention levels than with a negatively valenced one. Among the individuals not included in the at-risk groups, those who read the warning messages with vague target groups had a higher third-person perception toward smog risk than those targeting specific population groups. Practical and theoretical implications are discussed.

## 1. Introduction

Since the Industrial Revolution and the subsequent population growth, air pollution has become an increasingly severe environmental problem, responsible for roughly 5 million deaths worldwide each year [1]. This type of risk may not be perceptible by laypeople [2], and it was not until 1976 that the Pollutant Standards Index (PSI) was finally established to rate ambient air quality conditions by the US Environmental Protection Agency (EPA) [3]. In 1999, the EPA sought to communicate the health risks of air quality conditions more clearly to the public, and so the agency replaced the PSI with the Air Quality Index (AQI), which has been used internationally ever since [4]. The AQI updated the calculation of the PSI and categorized air quality conditions based on the National Ambient Air Quality Standards [5]. Each AQI category is named with a simple descriptor, such as “Good” for an index from 0 to 50 or “Moderate” for an index from 51 to 100 [6]. In 2000, based on epidemiological evidence of air pollution’s adverse health effects on different population groups, an updated version of the Air Quality Guidelines from the World Health Organization added warning messages for different population groups at different AQI levels [7]. Using descriptors and warning messages for different population groups has become standard in the AQIs of many countries.

China has some of the worst air pollution in the world [8]. In response to this crisis, the Chinese government began building a 24-h AQI monitoring system for its major cities in 2012 [9]. Nevertheless, despite efforts from the government and civil organizations to communicate the harms of smog to the public, the public has been less responsive than expected. According to an online survey conducted in 2018 by the Dongcheng District Center for Disease Control and Prevention (CDC) in Beijing, 84.1% of the 15,334 participants believed that smog was harmful to health, but only 50.9% wore air-filtering masks in the smog [10]. This contradiction raises a red flag and invites further investigation.

The communication of risk information plays a fundamental role in health promotion. However, as a primary mass communication method for governments to inform the public of the risks of smog, the effect of AQI and its health messages have not been noticed by researchers from the communication domain. Recent studies investigating the media content of environmental risk mainly focused on socially constructed messages [11,12,13], and only a few considered how the public interpreted and understood the air quality information [14]. To our knowledge, no prior research empirically validated the psychological effect of AQI information. Besides, we discovered considerable variations in information design by overviewing the AQIs from 11 countries (regions). Although AQI is designed to achieve comprehensiveness among the public in each society, different forms of information may open up room for unintended interpretation, which further shape the public’s risk perception and subsequent precaution against smog. After all, individuals react to the hazards they perceive. If their perceptions are faulty, “efforts at personal, public and environmental protection are likely to be misdirected” [15] (p. 137). Therefore, to address the inefficiency of some styles of AQI information in promoting the public’s precaution against bad air, an experiment was conducted employing factual AQI information used in different countries/regions as examples.

The study would provide a psychological approach to comprehend the AQI information in different formats. Then, the experimental results would contribute to our knowledge of how the public perceives environmental health risks through scientific information and help better design such risk information for health promotion.

## 2. Literature Review

### 2.1. AQI Information

Classifying air quality into different conditions is a readily comprehendible way to guide the public’s perceptions of the health risks of air pollution [16]. However, mixed epidemiological evidence has indicated that these classifications may be problematic. First, experts have not reached a consensus on what constitutes a “safe level” on the AQI [16,17] (p. 173). When predicting the mortality rate of particulate matter (PM, one of the major pollutants in the air), a linear model is most frequently reported [3,18,19,20]. Long-term exposure to ambient air pollution with PM2.5, even at a low concentration (e.g., 10 μg/m^3^), is significantly related to a higher lung cancer incidence rate and a higher cardiopulmonary mortality rate [21,22]. However, for practical reasons, the minimal 24-h PM2.5 concentration has been set to 30 μg/m^3^ in China and India, two times higher than the suggested value in the WHO guidelines [9,16,23]. It may be inappropriate to set a unified AQI across the globe [24].

The second edition of the WHO guidelines stressed the adverse effects of air pollution on sensitive and vulnerable groups [7]. Research showed that the elderly, children, and people with preexisting cardiovascular and respiratory conditions might experience more adverse health effects from smog exposure [18,25,26]. Besides, economic factors matter as well. Those with lower socioeconomic status or from developing countries have more risks from air pollution [27,28,29]. In 2017, half of the deaths caused by air pollution were in China and India, the world’s two most populous developing countries, while the air pollution mortality rate in high-income countries was below the global average [30].

AQI descriptors and warning messages vary across countries and regions (see examples in Table 1). For the first level of the AQI (0–50, i.e., the best air quality), “excellent” is used in mainland China while “good” is used in the US and India. “Good” in the Chinese AQI reflects the second level, which is coded as “moderate” in the US and India. The code “moderately polluted” is the fourth level in mainland China, which is equivalent to the “unhealthy” level in the US. Moreover, there are two styles of expression for the descriptors. The descriptors in the UK and Hong Kong are expressed in a relatively neutral manner, such as “low”, “moderate”, “high”, and “very high”. However, most countries, including South Korea, India, Australia, and the US, use valenced words (i.e., words with affective features of both positive and negative emotions [31]), such as “good”, “unhealthy”, and “hazardous” [6,9,23,32,33,34,35].

There are also differences in the population groups to which warning messages are addressed. The warning messages in Canada, Mexico, the UK, and the EU are provided separately for two different population groups: the “at-risk/sensitive groups” and the “general population” [33,36,37,38]. In Hong Kong and Singapore, the broad “at-risk/sensitive groups” category is replaced with specified individual groups, such as “outdoor workers” or “the elderly, pregnant women, and children” [35,39]. In mainland China, India, South Korea, and Australia, the warning messages targeting the “at-risk/sensitive groups” and the “general population” are presented along with specific individual groups, such as “children”, “patients”, and “people with asthma” [6,9,23,32,34]. Hence, the specific at-risk groups mentioned in AQI warning messages are not uniformly defined and remain ambiguous to some degree.

To sum, no “safe” level of air pollution exists. The responsibility of taking precautions against bad air falls on each individual’s shoulders based on their perception of such risk. Notably, the elements of AQI information take various forms across the world. Hence, the efficiency of the different forms of AQI information in promoting precaution actions needs to be scrutinized, even though that information is primarily based on scientific facts.

Based on Table 1, we classified the current AQI descriptors into two categories, “neutral (e.g., moderate, low, and high)” and “valenced (e.g., unhealthy, bad, and hazardous)”. Furthermore, the population groups addressed in warning messages are either “specific (e.g., children, seniors, pregnant women, and people with respiratory diseases)” or the “vague (e.g., most people, sensitive and healthy population, and others)”. To better understand the influence of these different types of AQI descriptors and warning messages, we discuss the psychological effects of AQI information elements on risk perception and precaution intention below.

### 2.2. Valence of AQI Descriptor and Risk Perception

Risk perception is typically subjective. Earlier research has identified three factors contributing to individuals’ risk perception: severity, high technology, and the number of people exposed [15,40]. The severity dimension has been later conceptualized as the degree of dread in a two-dimension model of risk perception in science [41]. The dread risk refers to uncontrollable, fatal, involuntary, and catastrophic outcomes [42]. However, given that risk is a social construct [43], the level of dread is subject to individuals’ reactions to risk information, especially when the information is in different styles.

Compared with the AQI descriptors of “low” or “high”, valenced words such as “good” and “hazardous” usually provide more room for emotional activation. Valence refers to “the direction of behavioral activation associated with emotion, either toward (pleasant emotion) or away from (unpleasant emotion) a stimulus” [44] (p. 990). Slovic and Peters have suggested that strong visceral emotions play a vital role in shaping the public’s self-risk perception [45]. Indeed, the relationship between valenced information and self-risk perception has been validated. For instance, Hornsey and Fielding compared the effects of neutral, optimistic, and pessimistic messages about reducing global carbon emissions and found that those who read pessimistic messages had higher levels of risk perception than those who read optimistic and neutral ones [46]. By the same token, we expect that AQI with negatively valenced descriptors may be associated with higher levels of self-risk perception than neutral ones.

Risk perception is a crucial antecedent of health behavior [47]. The relationship between self-risk perception and subsequent precaution intention has been widely validated [48]. For example, Van der Weerd and colleagues found that the intention to receive the H1N1 influenza vaccination was associated with the public’s perceived fear and worry over H1N1 [49]. Similarly, individuals with a higher self-risk perception of smog are more inclined to take precautions against smog.

Therefore, the following hypotheses are postulated:

**Hypothese** **1** **(H1).** *Individuals who view a negatively valenced AQI descriptor will have higher levels of self-risk perception of smog than those who view a neutral descriptor.*

**Hypothese** **2** **(H2).** *Individuals who view a negatively valenced AQI descriptor will have higher levels of precaution intention against smog than those who view a neutral descriptor.*

**Hypothese** **3** **(H3).** *Self-risk perception of smog will mediate the relationship between the valence of descriptor and precaution intention against smog.*

### 2.3. Third-Person Effect of AQI Warning Messages

The third-person effect (TPE) suggests that people tend to overestimate the impact of negative mass communications on others in terms of attitudes and behavior [50]. There are two key components of TPE: The first is the perceptual component—the third-person perception (TPP)—focusing on the perception gaps of “how much influence media content may have on the self versus others”, and the behavior component refers to “the real-life consequences that may result from these perception gaps” [51] (p. 540).

Prior studies investigating the TPP in terms of risk perception have confirmed the pattern that people significantly perceive less risk to themselves than others. For instance, Chen and Atkin investigated TPE in the context of Internet privacy risks and found that users reported greater risk perception to others than to themselves [52]. This biased optimism was also discovered when facing health risks such as cancer [53] and avian flu [54]. By the same token, when receiving AQI information indicating hazardous outcomes of smog, an overall tendency is expected that people will perceive less risk to themselves than others.

Researchers have argued that the illusionary bias of invulnerability may result from the downward comparison with a vague and distant other [55]. In other words, TPP may be enhanced as the psychological distance and the vagueness between oneself and others in comparison increase [56]. Harris and Middleton discovered that subjects displayed more optimistic bias when compared with a vague average other than with a specific friend [57]. It is likely that the vague expression of the “average person” reminded participants of someone who is “less advantaged, less intelligent and generally worse off than the self” [58] (p. 503). The mechanism could also be explained by the proximity of one’s psychological distance, as people generally respond less strongly to psychologically distant others (e.g., a stranger in another country) than to those who are close to them (e.g., someone they know in their community) [59]. After comparing both effects of vagueness and closeness, Duck and Mullin suggested that the TPP occurred particularly in the vague and distant condition when receiving negative content [55].

In the context of AQI information, compared with the specific indication such as “children, seniors and people with respiratory diseases”, the alternative use of the “sensitive/at-risk population” has a higher level of vagueness. The vague target groups such as “sensitive groups” may induce people to automatically choose a vague person who is more vulnerable than themselves. In contrast, the specific target group, such as “children, seniors, and people with respiratory diseases”, provides a specific comparison target. As a result, for the people who do not belong to any of the at-risk population groups (i.e., healthy young adults), the vague target groups will elicit more biased risk perception between self and others than using the specific target groups.

Moreover, prior evidence has revealed that TPP may lead to behavioral consequences such as supporting the censorship of pornography and online misinformation [60,61,62]. Apart from the restriction behavior induced by TPP, TPP may also promote compliance intentions [53,63]. For instance, by examining the connection between TPP and behavioral responses toward the avian flu news coverage, results showed that TPP might reduce respondents’ intention to seek information about the flu and subsequent behavior intention to get vaccinated [54]. Similarly, Stavrositu and Kim discovered a negative association between TPP and behavioral intentions [53]; in other words, people with strong perception bias toward social media metrics of the cancer story have less interest in taking preventive measures against cancer. Hence, it is logical to predict an inverse relationship between TPP of smog risk and precaution intention.

Taken together, the following hypotheses are postulated:

**Hypothese** **4** **(H4).** *Individuals will perceive less risk of smog for themselves than for others.*

**Hypothese** **5** **(H5).** *Individuals who are not in the at-risk groups will have more third-person perceptions of the smog risk when the target groups in warning messages are vague rather than specific.*

**Hypothese** **6** **(H6).** *Individuals’ third-person perception of the smog risk is negatively related to their precaution intention against smog.*

## 3. Method

### 3.1. Procedure

**Figure 1:**
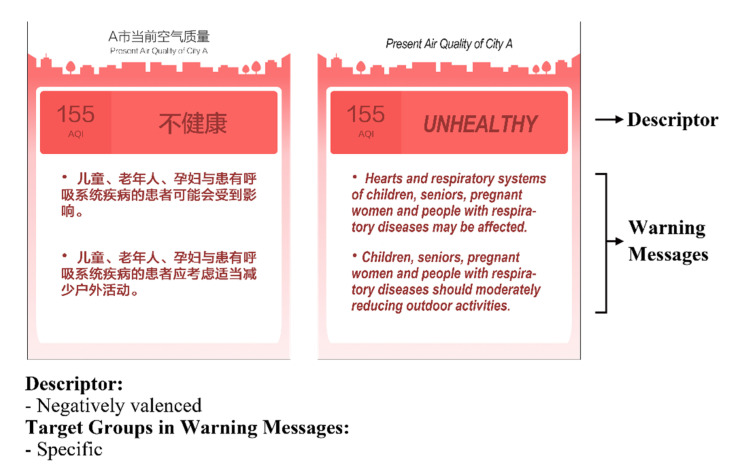
The layout of the experimental stimuli.

A between-subject experiment with a 2 (descriptor: neutral vs. negatively valenced) × 2 (target groups in AQI warning messages: vague vs. specific) factorial design was conducted online in mainland China. Subjects were randomly assigned to one of four conditions. In each condition, the subjects were asked to imagine that they had just arrived at City A and were checking the current air quality condition with their mobile phone. A fictitious AQI notification page was presented (see example in Figure 1). The stimuli format was a typical AQI notification from the mobile phone application *AirVisual*, which was the top-ranked air quality app on both Google Play and the Apple App Store download list at the time of writing.

All participants formally consented before experimenting. After viewing the stimuli, the subjects were asked to fill out a questionnaire measuring their self-risk perceptions of themselves and others, respectively, and their intention to take precaution actions. The subjects’ knowledge levels of smog were also measured as a control variable. The participants took less than half an hour to complete the online experiment. Once the subjects finished the questionnaire, they were debriefed and thanked.

### 3.2. Stimuli

As Figure 1 suggests. The manipulation of descriptor and target groups in AQI warning messages was based on an AQI of 155. It was the average AQI level in five major Chinese cities (Beijing, Shanghai, Guangzhou, Chengdu, and Shenyang) for the winters of 2015 to 2019 [30]. Besides, this level of AQI suggests considerable health risks, especially for at-risk groups.

The content of the experimental stimuli was based on factual AQI information from various countries or regions. The descriptor was labeled as either “moderately polluted” (neutral, as used in countries like mainland China and India) or “unhealthy” (negatively valenced, as used in countries like the US and South Korea). As for the vagueness of target groups, in the vague condition, the warning message was worded as “Hearts and respiratory systems of sensitive individuals may be affected. Sensitive individuals should consider moderately reduce outdoor activities” (as used in countries like the US). In the specific condition, the warning message was, “Hearts and respiratory systems of children, seniors, pregnant women, and people with respiratory diseases may be affected. Children, seniors, pregnant women and people with respiratory diseases should consider moderately reduce outdoor activities” (as used in countries like Singapore).

### 3.3. Sample

A priori power analysis using G*Power 3.1 [64] was conducted for sample size estimation. Since no previous relevant research investigating the effect of AQI information was available, we chose the closest study examining the effect of different environmental news frames on participants’ risk perception by Durfee [65], and the effect size Cohen’s d = 0.64. With an alpha level of 0.05, powered at 80%, a total sample size of 79 was required to detect a significant effect.

One hundred and seventy-seven participants were recruited from Survey Star (Wen Juan Xing), one of China’s largest commercial survey platforms. Survey Star was contracted to send a recruitment announcement to its national sampling pool of adults. All participants were compensated with bonus points, accumulated, and exchanged for cash or consumer products. After the manipulation check and the removal of participants with relevant preexisting health conditions (e.g., respiratory diseases), 150 valid responses were included in the data analysis. Hence, enough power was ensured in the analysis. Among them, there were 68 females (45.3%) and 82 males (54.7%). The average age was 29.65 years (*SD* = 7.52). The average monthly income level was between RMB 4001 (around US 600) and RMB 6000 (around US 900). In terms of education level, 6.6% of the participants had received a high school or lower education, 94% had a bachelor’s or other college degree, and 6% had a post-graduate degree or higher.

### 3.4. Measures

*Self-risk perception of smog* was measured by four questions modified from Cheng and colleagues [66], including, “How likely is your (1) physical health, (2) mental state, (3) work/study performance, and (4) daily life going to be affected by the present air quality?” The same question asked *risk perception for others* with a different comparison target of “an average person of your age.” Subjects were asked to rate each item on a 5-point Likert scale ranging from 1 (*strongly disagree*) to 5 (*strongly agree*). The reliability coefficient of Cronbach’s alphas were 0.81 for self-risk perception and 0.84 for risk perception of others, respectively.

*The third-person perception* was calculated by subtracting the respondents’ self-risk perception from their risk perception for others.

*Precaution intention* was measured based on the scale of protective actions against air pollution developed by Wei and colleagues [67], including “wearing an air-filtering mask outdoors”, “closing windows and reducing outdoor activities”, “using air purifier indoors”, “avoiding going to the high-traffic area of the city”, and “leaving this city as soon as possible”. Those question items were modified into a 5-point Likert scale to detect subjects’ precaution intentions from 1 (*strongly disagree*) to 5 (*strongly agree*). The reliability coefficient of Cronbach’s alpha was 0.72.

*Knowledge of smog* was used as a control variable. The original measures were developed by environmental scientists and journalists [68]. Nine true-or-false questions were used to capture three aspects: the harm of smog (e.g., “Smog can cause global warming,” False), contributors of smog (e.g., “The main components of smog are sulfur dioxide, nitrogen oxides (gaseous), and inhalable particulate matter,” True), and approaches to control smog (e.g., “Raising China’s oil standards can alleviate the smog problem in some parts of China,” True). The sum of correct answers was calculated as each participant’s knowledge level of smog.

The participants’ demographic variables, including sex, age, income, and education level, were gauged with conventional measures. 

The full text of the questionnaire is available in Supplementary Material File S1.

### 3.5. Attention and Manipulation Check

Survey Star provides standard questions for checking respondents’ attention so that the responses of participants who complete the questions too quickly or carelessly can be removed. To further ensure the success of the manipulation, the participants were given a multiple-choice question to help them recall the content of the AQI descriptors and warning messages immediately after reading the stimuli. If they gave an incorrect answer or chose “can’t remember”, they were removed from further analyses.

## 4. Results

### 4.1. Descriptive Results

The means, standard deviations, and inter-correlation for the key variables are shown in Table 2. Among all the 150 subjects, the average score of the nine true-or-false questions for testing the respondents’ prior knowledge of smog was 5.37 (*SD* = 1.78), and there was no significant difference across all four conditions (*F*(3, 146) = 1.40, *p* = 0.25). Overall, the participants had a moderate level of risk perceptions for themselves (*M* = 3.38, *SD* = 0.76) and for others (*M* = 3.47, *SD* = 0.78). The average precaution intention was 4.02 (*SD* = 0.60).

### 4.2. Hypotheses Testing

Two-way analyses of variance (ANOVA) were conducted to test the effects of the different uses of descriptors and target groups in AQI warning messages on subjects’ self-risk perception, TPP, and precaution intention to reduce type I error caused by conducting a series of *t*-tests (Table 3). For subjects’ self-risk perception toward smog, results indicated that those who read neutral descriptors perceived less risk than did those who read negatively valenced descriptors (*F*(1, 146) = 32.54, *p* < 0.001, *η_p_^2^* = 0.18). Hence, H1 was supported.

For precaution intention against smog, results showed that subjects in the neutral descriptor condition had less precaution intention than those in the negatively valenced group: *F*(1, 146) = 17.28, *p* < 0.001, *η_p_^2^* = 0.11. Therefore, H2 was supported.

A paired-sample *t*-test indicated that the participants’ self-risk perception (*M* = 3.38, *SD* = 0.76) was significantly lower than risk perception of others (*M* = 3.47, *SD* = 0.78): *t*(149) = −2.68, *p* = 0.008. Simply put, an overall tendency of TPE occurred. Thus, H4 was supported.

Subjects who received warning messages targeting vague population groups had higher levels of TPP of smog risk than those who received messages targeting specific groups: *F*(1, 146) = 4.84, *p* = 0.029, *η_p_^2^* = 0.03. Therefore, H5 was also supported.

**Figure 2:**
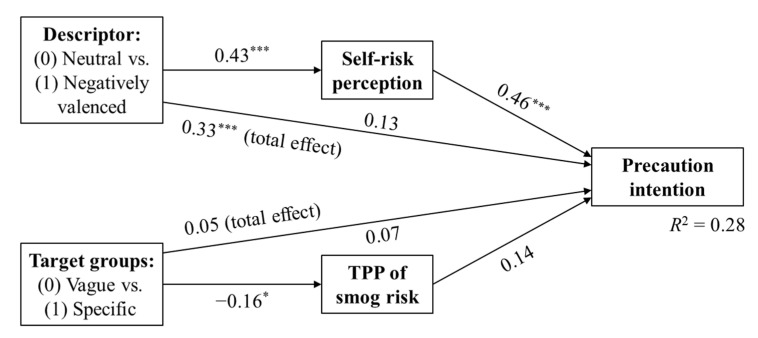
Path model results. Note: * *p* < 0.05 (significance level), *** *p* < 0.001.

To test H3 and H6 and obtain a panoramic picture of the proposed model, we conducted path modeling using the maximum likelihood estimator to test the relationships among variables and mediating effects of self-risk perception and TPP. Descriptor and target groups in warning messages were used as the exogenous variables, and all other variables were modeled as endogenous variables (see Figure 2). The model fit has reached an acceptable level: χ^2^/*df* = 1.61, *p* = 0.20, CFI = 0.99, RMSEA = 0.06.

Results of mediation analysis confirmed a full mediation effect of self-risk perception on the relationship between the valence of descriptor and precaution intention. The total effect was *β* = 0.33, *p* < 0.001. AQI descriptor had no direct effect on subjects’ precaution intention against smog (*β* = 0.13, *p* = 0.088), but was indirectly positively mediated by their self-risk perception (*β* =0.43, *p* < 0.001), which positively associated with precaution intention (*β* = 0.46, *p* < 0.001). Those results supported H3 and further confirmed H2.

However, the mediation effect of TPP on the relationship between target groups in waning messages and precaution intention was insignificant: *β* = 0.05, *p* = 0.472. Specifically, different use of target groups in warning messages was not associated with precaution intention (*β* = 0.07, *p* = 0.297), though a significant indirect effect was found on TPP toward smog risk (*β* = −0.16, *p* = 0.044). And TPP was not associated with precaution intention (*β* = 0.14, *p* = 0.054). Thus, H6 was not supported, but those results lent support to H5. 

The raw data of this study are available in Supplementary Material File S1. 

## 5. Discussion

As an “intangible” risk that has existed since the beginning of the Industrial Revolution, smog was not codified into an index until the establishment of the PSI in the 1970s. Its revised version, the AQI, is an essential tool for governments worldwide to inform the public about air quality and recommend countermeasures. According to the latest guidelines for air pollution from WHO, specific thresholds for different pollutants and corresponding warning messages have been made for feasible and practical purposes, especially for developing countries that lack the ability to conduct their own impact assessment [16,18]. While different styles of AQI information exist in various countries or regions, the nuance of AQI elements may influence the public’s cognitive and behavioral reactions to poor air conditions. Indeed, the present study discovered some potential deficiencies in some designs of AQI descriptors and the warning messages addressing at-risk groups.

First, this study has revealed that the styles of AQI descriptors influenced participants’ perceptions and precaution intentions. The results indicate that neutral descriptors are associated with lower levels of risk perception toward smog and subsequent precaution intentions against smog than negatively valenced descriptors. Besides, in terms of warning messages, the participants who read the messages with vague target groups (e.g., “sensitive individuals”) exhibited a higher level of TPP toward smog risk than those who read messages with specific target groups (e.g., “children, seniors, pregnant women and people with respiratory diseases”). In other words, a higher level of vagueness in the expression of at-risk population groups in AQI information may elicit more biased optimism among the public that they are less likely influenced by smog than others.

However, we did not find any correlation between TPP and precaution intention, in contradiction to the link between the perceptual and behavioral components of TPE. Previous studies discovered that participants who received statistical health messages perceived more risk than those who read qualitative risk information [69]. Similarly, descriptors with a statistical air quality index and larger font size may be more effective than qualitative warning messages [70]. Therefore, the descriptors in AQI may have played a more crucial role than targeting different population groups in the warning messages in affecting short-term precaution intention.

Theoretically, this study sought to establish an interdisciplinary dialogue between risk communication and environmental science. It is generally agreed that all science consists of a degree of uncertainty [71,72], and it involves lots of controversy on communicating uncertainty to the public [73]. As a tool that translates the scientific and technical air pollution information into lay language for the public, AQI “must be made using [the] best information available” [16] (p. 179). Nevertheless, the “best” might be a relative concept. By analyzing the communicable elements of AQI and utilizing the theories in social psychology, the present study indicated the deficiencies of some AQI information styles and offered corresponding suggestions from the perspective of health communication.

Practically, by analyzing the effects of elements of the AQI, the present study highlights the following implications. First, to promote higher levels of precaution intention toward smog, negatively valenced descriptors (such as “unhealthy” and “hazardous”) are preferred to neutral ones. Policymakers from countries such as Canada appear to have realized the pitfall of using the descriptors “low, moderate, or high” and have modified the Air Quality Health Index standards to say “low risk, moderate risk, and high risk” for disambiguation [74]. However, at the time of writing, regions like the UK and EU continue to use neutral descriptors, and other countries (such as South Korea and Singapore) mix AQI descriptors, using both neutral and negatively valenced words. Those approaches may not be the most effective. Besides, specifying those exact at-risk groups who have a higher chance of getting affected by smog may be a better practice for clarification and minimizing the intensification of TPE.

### Limitations and Future Research

This study has several limitations. First, a common drawback of experimental studies is that the stimuli cannot include all possible combinations of manipulated variables. Future studies should consider the impact of other levels of AQI, such as “good” or “hazardous” levels. Future research may extract more features, such as the effect of different sub-groups of sensitive groups in Table 1, from AQI information worldwide to further investigate risk perception and subsequent precaution intention.

Second, the major limitation of the study was the generalizability of the results. The experiment was conducted among Chinese netzines and the sample comprised mainly healthy young adults, and we purposely excluded those from at-risk groups, which may undermine the external validity of the present study. Public reactions to AQI information depend on various factors. For at-risk groups, such as the elderly and people with illnesses, their reactions may be different. Future studies should scrutinize the features of warning messages emphasizing sensitive groups and tested with different subjects or in a different cultural setting.

Third, the measures of participants’ risk perception in the present study were based on the classical two-dimension model by Slovic [41]. Future research may use different measurements (such as the recent CoWoRP scale of four-dimension risk perception: probability, severity, worry, and unsafe by Man et al. [75]) to gauge the participants’ perceived risk toward smog.

Last, smog is a time- and location-based risk. The online experiment in the present study asked participants to imagine a situation in which they would encounter smog, which may not reflect the real-world situation, given each participant is in different places and the present air condition may vary significantly. The external validity of the study was compromised this way. Future research may consider offline experiments in different locations and countries to gain more validity in different cultures.

## 6. Conclusions

This study adds an overlooked but crucial angle to the growing knowledge of communicating environmental health information to the public about the effects of different styles of AQI information on individuals’ risk perception and precaution intention. As predicted, neutral AQI descriptor and warning messages with vague target groups were found less efficient in shaping viewers’ self-risk perception of smog and subsequent precaution intention relative to the negatively valenced descriptor and warning messages with specific target groups. Those findings shed light on the better design of AQI information to urge the public to take precautions against air pollution. As Fischhoff and Davis suggested [71], there are two types of objectives in scientific communication: non-persuasive and persuasive. Providing people choices that best serve their interest is the purpose for the non-persuasive objective. While for the persuasive objective, reducing uncertainty is desirable. The issue of communicating air pollution information features both objectives. Scientific accuracy and better communication effect should not contradict each other. The present study suggests the potential room for improving AQI design while maintaining scientific merit.

## Figures and Tables

**Table 1:** Examples of the descriptors and target groups in warning messages of AQI from 11 countries/regions.

Countries/Regions	AQI Types	Level, Descriptor, Index Range	Target Groups in Warning Messages at Each Level
Mainland China [9]	AQI (Air Quality Index)	1, Excellent, 0–50 2, Good, 51–100 3, Lightly Polluted, 101–150 4, Moderately Polluted, 151–200 5, Heavily Polluted, 201–300 6, Severely Polluted, >300	(1) All population (1) (2) Hypersensitive population (2) (3) Healthy population (3–6) (4) Sensitive population (3, 4) (5) Children and seniors (3–6) (6) Individuals with respiratory or heart diseases (3–5) (7) Individuals with heart or lung diseases (5) (8) General population (4–6) (9) Sick people (6)
US [6]	AQI (Air Quality Index)	1, Good, 0–50 2, Moderate, 51–100 3, Unhealthy for Sensitive Groups, 101–150 4, Unhealthy, 151–200 5, Very Unhealthy, 201–300 6, Hazardous, 301–500	(1) Unusually sensitive people (2) (2) Sensitive groups (3, 4) (3) General public (3, 4) (4) Everyone (5, 6)
South Korea [32]	CAI (Comprehensive Air-quality Index)	A, Good, 0–50 B, Moderate, 51–100 C, Unhealthy, 101–250 D/E, Very Unhealthy, 251–500	(1) Patients (all) (2) Sensitive groups (C–E) (3) General public (C, E)
Canada [37]	AQHI (Air Quality Health Index)	1–3, Low Risk, (Each^*^) 4–6, Moderate Risk, (Each^*^) 7–9, High Risk, (Each^*^) 10–12, Very High Risk, (Each^*^)	(*Separate warning messages for each population group*) (1) At-risk population (2) General population
India [23]	AQI (Air Quality Index)	1, Good, 0–50 2, Satisfactory, 51–100 3, Moderately Polluted, 101–200 4, Poor, 201–300 5, Very Poor, 301–400 6, Severe, 401–500	(1) Sensitive people (2) (2) People with lungs, asthma, and heart diseases (3) (3) Most people (4) (4) Healthy people (6) (5) Those with existing diseases (6)
Hong Kong [35]	AQHI (Air Quality Health Index)	1–3, Low, (Each^*^) 4–6, Moderate, (Each^*^) 7, High, (Each^*^) 8–10, Very High, (Each^*^) 10+, Serious, (Each^*^)	(Separate warning messages for each population group) (1) People with existing heart or respiratory illnesses (2) Children and the elderly (3) Outdoor workers (4) General public
EU [36]	EAQI (European Air Quality Index)	1, Good, (Each^*^) 2, Fair, (Each^*^) 3, Moderate, (Each^*^) 4, Poor, (Each^*^) 5, Very Poor, (Each^*^) 6, Extremely Poor, (Each^*^)	(Separate warning messages for each population group) (1) Sensitive population (2) General population
UK [33]	DAQI (Daily Air Quality Index)	1–3, Low, (Each^*^) 4–6, Moderate, (Each^*^) 7–9, High, (Each^*^) 10, Very High, (Each^*^)	(Separate warning messages for each population group) (1) At-risk individuals (2) General population
Australia [34]	AQI (Air Quality Index)	1, Very Good, 0–33 2, Good, 34–66 3, Fair, 67–99 4, Poor, 100–149 5, Very Poor, 150–200 6, Hazardous, >200	(1) Sensitive groups (6) (2) Others (3) (3) Other adults (4–6) (4) Anyone who experience symptoms (4)
Mexico [38]	AIR AND HEALTH Index (Air Quality and Health Risks Index)	1, Good, (Each^*^) 2, Regular, (Each^*^) 3, Bad, (Each^*^) 4, Very Bad, (Each^*^) 5, Extremely Bad, (Each^*^)	(Separate warning messages for each population group) (1) Sensitive groups (2) For the entire population
Singapore [39]	PSI (Pollutants Standards Index)	1, Good, 0–50 2, Moderate, 51–100 3, Unhealthy, 101–200 4, Very unhealthy, 201–300 5, Hazardous, >300	(Separate warning messages for each population group) (1) Healthy persons (2) Elderly, pregnant women and children (3) Persons with chronic lung disease, heart disease

Note: Each * means that index boundaries depend on each pollutant without an overall index.

**Table 2:** Descriptive statistics and correlations among key variables.

	M (SD)	1	2	3	4	5	6
1. Descriptor (IV)	0: neutral; 1: negatively valenced	1					
2. Target groups (IV)	0: vague; 1: specific	0.01	1				
3. Self-risk perception	3.38 (0.76)	0.43 **	0.10	1			
4. TPP of smog risk	0.09 (0.39)	−0.02	−0.18 *	−0.20 *	1		
5. Precaution intention	4.02 (0.60)	0.33 **	0.10	0.50 **	0.03	1	
6. Knowledge of smog	5.37 (1.78)	−0.07	−0.15	−0.04	0.18 *	0.03	1

Note: (1) * *p* < 0.05 (significance level), ** *p* < 0.01; (2) IV for abbreviation of independent variable; (3) the IVs were dummy coded as dichotomous variables for analysis.

**Table 3:** Results of two-way ANOVA for the effects of the independent variables.

	Mean					
	Descriptor				Target Groups	
Variables	Neutral (*n* = 75)	Negatively valenced (*n* = 75)	F	Vague (*n* = 77)	Specific (*n* = 73)	F
Self-risk perception	3.07 (0.08)	3.71 (0.08)	32.54 ***	3.32 (0.08)	3.46 (0.08)	1.63
TPP of smog risk	0.09 (0.05)	0.08 (0.05)	0.06	0.15 (0.04)	0.01 (0.05)	4.84 *
Precaution intention	3.83 (0.07)	4.22 (0.07)	17.28 ***	3.97 (0.07)	4.08 (0.07)	1.40

Note: (1) * *p* < 0.05 (significance level), *** *p* < 0.001; (2) standard deviation was reported in the parenthesis.

## Data Availability

Data is contained within the supplementary material.

## Supplementary Materials

The following are available online at www.mdpi.com/article/10.3390/ijerph181910542/s1, (1) The data presented in this study are available in the CSV file; (2) The full text of the questionnaire was provided.
